# Inpatient treatments for adults with anorexia nervosa: a systematic review of literature

**DOI:** 10.1007/s40519-024-01665-5

**Published:** 2024-05-20

**Authors:** Federica Toppino, Matteo Martini, Paola Longo, Inês Caldas, Nadia Delsedime, Raffaele Lavalle, Francesco Raimondi, Giovanni Abbate-Daga, Matteo Panero

**Affiliations:** 1 Department of Neuroscience “Rita Levi Montalcini”, Eating Disorders Center, University of Turin, Via Cherasco 11, 10126 Turin, Italy; 2 Centro Hospitalar Psiquiátrico de Lisboa, Lisbon, Portugal

**Keywords:** Anorexia nervosa, Inpatient, Hospitalization, Treatment, Eating disorders

## Abstract

**Purpose:**

Anorexia nervosa (AN) is a mental disorder for which hospitalization is frequently needed in case of severe medical and psychiatric consequences. We aim to describe the state-of-the-art inpatient treatment of AN in real-world reports.

**Methods:**

A systematic review of the literature on the major medical databases, spanning from January 2011 to October 2023, was performed, using the keywords: “inpatient”, “hospitalization” and “anorexia nervosa”. Studies on pediatric populations and inpatients in residential facilities were excluded.

**Results:**

Twenty-seven studies (3501 subjects) were included, and nine themes related to the primary challenges faced in hospitalization settings were selected. About 81.48% of the studies detailed the clinical team, 51.85% cited the use of a psychotherapeutic model, 25.93% addressed motivation, 100% specified the treatment setting, 66.67% detailed nutrition and refeeding, 22.22% cited pharmacological therapy, 40.74% described admission or discharge criteria and 14.81% follow-up, and 51.85% used tests for assessment of the AN or psychopathology. Despite the factors defined by international guidelines, the data were not homogeneous and not adequately defined on admission/discharge criteria, pharmacological therapy, and motivation, while more comprehensive details were available for treatment settings, refeeding protocols, and psychometric assessments.

**Conclusion:**

Though the heterogeneity among the included studies was considered, the existence of sparse criteria, objectives, and treatment modalities emerged, outlining a sometimes ambiguous report of hospitalization practices. Future studies must aim for a more comprehensive description of treatment approaches. This will enable uniform depictions of inpatient treatment, facilitating comparisons across different studies and establishing guidelines more grounded in scientific evidence.

**Level of evidence:**

Level I, systematic review.

**Supplementary Information:**

The online version contains supplementary material available at 10.1007/s40519-024-01665-5.

## Introduction

Anorexia nervosa (AN) is a severe mental disorder characterized by an intense fear of gaining weight and a distorted body image. Individuals with AN exhibit severe dietary restrictions or engage in other weight loss behaviors such as excessive physical activity or purging, resulting in low body weight. The medical complications of AN affect various organs and systems and are accompanied by impairments in cognitive and emotional functioning [1, 2]. These severe symptoms often require intensive treatments such as hospitalization, partial hospitalization, and intensive ambulatory settings [3].

Inpatient treatment represents the highest level of care, especially for medically or mentally unstable patients or those unresponsive to outpatient care. AN is the eating disorder (ED) with the highest hospitalization rate, accounting for 32% of patients [4]. Intensive treatment in a hospital setting is usually determined by a set of clinical indicators [5]. Despite efforts with outpatient therapies, an increase in admission rates has been reported in several Western countries due to the inherent severity of the disease or resistance to outpatient care [6–8]. Weight gain is central to the treatment, with clinicians prescribing caloric intake while monitoring malnourished patients closely. Patient motivation is crucial, and psychological interventions during inpatient care aim to encourage the patient's willingness to change [1].

Several international guidelines, such as those from the World Federation of Societies of Biological Psychiatry (WFSBP) [9], National Institute for Health and Clinical Excellence (NICE) [10], Medical Emergencies in Eating Disorders (MEED) [11], and Royal Australian and New Zealand College of Psychiatrists Clinical Practice (RANZCP) [12], outline situations where hospitalization is appropriate. However, the criteria are not rigid and there is also room for the importance of clinical expertise and personalized therapy. Furthermore, there are some distinct national guidelines for the treatment of AN, such as a Spanish one from 2009 [13], a French one from 2010 [14], and a German one revised in 2018 [15]. One of the most recent is from the American Psychiatric Association (APA) and was published in February 2023 [16].

According to these guidelines, outpatient treatment is considered the first-line therapy setting for AN patients. Criteria for more intensive care levels, such as full-time hospitalization, are also included. All guidelines highlight the importance of deciding on hospitalization based on individual factors that are to be considered for patients who have not responded to outpatient care or are at high risk for medical complications, such as extremely low body mass index (BMI), behavioral aspects, vital signs, psychiatric comorbidity, and environmental aspects [16]. While a BMI below 15 kg/m2 in adults or < 70% median BMI in children defines extreme malnourishment, it is stressed that BMI alone is not the sole criterion for hospitalization.

Effective AN treatment involves a multidisciplinary team, typically including psychiatrists, dieticians, nurses, psychologists, and internal medicine physicians [1, 16, 17]. Differences in international guidelines highlight the varying compositions and emphases of such teams: APA [16] and WFSBP [9] focus on extensive medical monitoring, while NICE [10] includes occupational therapists and social workers.

The core aspect of inpatient treatment is nutritional rehabilitation, focused on weight gain through personalized dietary plans and refeeding practices. Guidelines consistently favor oral enteral nutrition over parenteral nutrition, which should only be used as a last resort [16, 18]. Concerning weight gain, while lacking standardized protocols, there is some consensus on weight gain of 0.5–1.4 kg/week during hospitalization [12–14, 16]. Despite variations in starting caloric prescriptions, personalized dietary plans are recommended; some guidelines provide specific recommendations regarding supplementation [12, 13, 16], while others suggest a general vitamin supplement [10]. Furthermore, supplementary treatment suggestions were provided, such as meal support and supervised physical activity [16]. One guideline expressly advised against the use of physical therapies, including electroconvulsive therapy and transcranial magnetic stimulation [10].

While psychotherapy stands out as a crucial component of treatment, only a few guidelines have recommended specific interventions during hospitalization. Notably, Cognitive-Behavioral Therapy (CBT) is endorsed by several guidelines [10, 12, 13, 16], followed by Family-Based Treatment (FBT). Psychodynamic Psychotherapy and Interpersonal Psychotherapy are mentioned but with less unanimous support [16]. Novel treatments addressing emotional difficulties are under study. Motivation to change is central and should be assessed in a transtheoretical model and with motivational interviewing. MEED guidelines also address work on the lack of insight and tendency to sabotage, emphasizing the importance of individual contracts with patients [11]. RANZCP underlines the role of family therapy [12].

Regarding medication, three guidelines [10, 12, 13] emphasize that it should not be the primary treatment. Almost all guidelines recommend fluoxetine as the preferred selective serotonin reuptake inhibitor (SSRI) [9, 12, 13, 16], either alone or in combination with psychotherapy [16], or fluvoxamine [9]. However, one guideline [15] discourages the use of antidepressants for weight gain.

The transition from inpatient to outpatient care is crucial for AN, with care plans specifying discharge and reintegration into community-based care [10, 19]. The end of inpatient treatment often results in care discontinuity, stressing the need for a smoother transition, supported by psychological interventions [20]. International guidelines vary in their follow-up recommendations: APA [16] and WFSBP [9] suggest regular and gradually tapering follow-up sessions, NICE [10] advocates for a named professional to coordinate comprehensive care plans, RANZCP [12] emphasizes early planning for discharge and continuous psychotherapy.

The present study aims to review the current scientific literature about hospitalization in AN, analyzing both the adherence to established guidelines and cases in which real-world practices differ from standards of treatment. We encountered a general paucity of comprehensive reviews on hospitalization, except for a previous work conducted by Suárez-Pinilla and colleagues in 2015 [21], which focused on treatment (especially psychopharmacology). Although the review has the merit of underlining some key aspects of the treatment, it is dated, considers only the randomized controlled trial (RCT) studies, does not specifically analyze the adult population, and does not consider some crucial factors of hospitalization. Unlike the paper from 2015, we aim to cover a broader examination, encompassing other aspects of inpatient treatment and including a more diverse set of studies. Additionally, our scope is to incorporate the most recent literature, extending from 2013 onwards.

Our intention is therefore to cover through the method of the systematic review a wide range of aspects related to hospitalization, addressing a gap in the current scientific literature and recognizing that it is not uncommon to venture beyond established guidelines in psychiatry. Our objective is to highlight the distinctions between routine treatment decisions and their corresponding established protocols. Despite the availability of treatment guidelines, there is no universally defined type A hospital treatment. Inpatient treatment is the preferred setting when other forms of outpatient care (e.g., ambulatory, day hospital) prove insufficient. In cases where AN reaches a severe stage, posing a threat to a patient's life, it becomes crucial to provide a secure environment for a comprehensive treatment.

## Methods

The following systematic review adheres to the criteria outlined in the Preferred Reporting Items for Systematic Reviews and Meta-Analyses—Extension for Scoping Reviews PRISMA-ScR statement [22]. The search spanned from June to October 2023 and encompassed databases such as PubMed, PsycInfo, Embase, and Cochrane Central Register of Controlled Trials. The search strategy involved the combined use of the terms “inpatient” OR “hospitalization” AND “anorexia nervosa”. The PRISMA checklist, the PRISMA checklist for abstracts, and the flow diagram of the studies included are available from Supplementary Materials.

The included papers adhere to the following criteria: (a) studies published between 2011 and October 2023; (b) studies investigating inpatient treatment in hospitals for AN (regardless of the type); (c) studies published in English. Single case studies, reviews, and studies on pediatric populations were excluded. Additionally, studies related to inpatient treatment in residential facilities were excluded. Neither the gender nor nationality of participants nor the absence of healthy controls served as exclusion criteria. In addition to keyword searching, we used citation chaining in full-text screening to intercept content that the original searches may have missed. The authors F.T., I.C., and M.P. individually conducted the assessments and later discussed any discrepancies regarding the articles and inclusion criteria. Out of the initial 846 studies identified, 105 were chosen for full-text review. Among these, 49 were excluded due to their predominant focus on pediatric populations [23, 24], 21 were omitted because they also pertained to non-hospitalization settings [25, 26] and 8 were disregarded for their inclusion of a significant number of patients with various EDs [27, 28]. Consequently, the final 27 studies were selected for this review (see Flow Diagram in Supplementary Materials). Four investigators (F.T., M.P., R.L., and F.R.) evaluated the methodological quality of the studies included using the 2018 version of the Mixed Methods Appraisal Tool (MMAT), developed by Hong and colleagues [29]. The overall agreement was 80%, and discrepancies were resolved through discussion. The list of the studies included, their characteristics and the results of the evaluation of the methodological quality are reported in Table 1.

**Table 1:** Article summary of study results included in this review

N	Authors	Sample size (*n*)	Diagnostic criteria	Diagnosis	Mean duration of illness (years)	Mean age (years)	Gender
1	Carter et al. [30]	138	DSM-IV	62% ANR, 38% ANBP	6.10	25.30	n.r
2	Money et al. [31]	28	DSM-IV	n.r	n.r	25	96.43% F, 3.57% M
3	Davies et al. [32]	Center 1: 46; center 2: 34	DSM-IV	n.r	10.6	26.1	n.r
4	Long et al. [33]	34	DSM-IV	29.41% ANR, 70.59% ANBP	13.00	33	100% F, 0% M
5	Mander et al. [34]	39	DSM-IV	n.r	8.60	27.70	97.1% F, 2.9% M
6	Pemberton et al. [35]	8	ICD-10	n.r	n.r	n.r	87.50% F, 12.50% M
7	Zuchova et al. [36]	34	n.r	n.r	Group 1: 8.20; group 2: 7.30	Group 1: 26.80; group 2: 25.47	97.06% F, 2.94% M
8	Hofer et al. [37]	65	DSM-IV	76.4% ANR, 23.26% ANBP	5.00	27.90	93% F, 7% F
9	Schlegl et al. [38]	435	ICD-10	72.6% ANR, 25.5% ANBP	8.84	26.36	100% F, 0% M
10	Andrade et al. [39]	169	DSM-5	60.9% ANR, 39.1% ANBP	3.68	22.61	94.08% F, 5.92% M
11	Hessler et al. [40]	770	ICD-10	39.6% ANR, 60.4% ANBP	n.r	27.79	100% F, 0% M
12	Marzola et al. [41]	137	DSM-5	67.6% ANR, 32.4% ANBP	7.10	24.90	100% F, 0% M
13	Chatelet et al. [42]	107	ICD-10	57.9% ANR	9.00	26.90	96.26 F, 3.74% M
14	Dittmer et al. [43]	207	DSM-IV	64.3% ANR, 16.1% ANBP, 19.6% AAN	Group 1: 4.07; group 2: 3.31	Group 1: 20.04; group 2: 18.32	100% F, 0% M
15	Gjoertz et al. [44]	107	ICD-10	57.6% ANR, 58.1% ANBP	10.40	Group 1: 27.60; group 2: 26.60	96.26% F, 3.74% M
16	Guarda et al. [45]	149	DSM-IV, DSM-5	42.2% ANR, 33.6% ANBP	n.r	34.93	89.9% F, 10.1% M
17	Guinhut et al. [46]	354	DSM-IV	48.8% ANR, 49.3% ANBP	9.50	28.70	95.76% F, 4.24% M
18	Yamazaki et al. [47]	188	DSM-IV, DSM-5	53.2% ANR, 46.8% ANBP	n.r	28.77	97.34% F, 2.66% M
19	Smith et al. [48]	75	DSM-IV	61.3% ANR, 38.7% ANBP	6.70	25.92	97.3% F, 2.7% M
20	Dauty et al. [49]	37	DSM-5	45.95% ANR, 54.05% ANBP	13.00	32.00	91.89% F, 8.11% M
21	Young et al. [50]	50	DSM-5	54% ANR, 46% ANBP	14.35	29.88	100% F, 0% M
22	Fouladi et al. [51]	Center 1: 38; center 2: 55	n.r	n.r	n.r	Group 1: 23.20; group 2: 27.70; group 3: 22.60; group 4: 27.40	n.r
23	Funayama et al. [52]	55	ICD-10	61.85% ANR	10.80	Group 1: 33.40, group 2: 35.90, group 3: 35.40, group 4: 36.10	97% F, 3% M
24	Hellwig-Walter et al. [53]	19	DSM-IV	78.95% ANR, 21.05% ANBP	9.00	23.79	100% F, 0% M
25	Hemmingsen et al. [54]	36	ICD-10	64% ANR, 36% ANBP	7.50	25.30	97.22% F, 2.78% M
26	Riedlinger et al. [55]	38	DSM-5, ICD-11	44.74% ANR, 42.11% ANBP	7.50	27.90	94.74% F, 5.26% M
27	Sjögren et al. [56]	49	ICD-10	n.r	6.47	24.30	93.88% F, 6.12% M

N	Authors	Follow-up	Mean length of stay	Employment	Admission criteria	Team
1	Carter et al. [30]	n.r	13.90 weeks	41% employed, 47% students, 12% unemployed	n.r	n.r
2	Money et al. [31]	n.r	n.r	n.r	n.r	n.r
3	Davies et al. [32]	n.r	n.r	n.r	n.r	n.r
4	Long et al. [33]	Outpatient care	47.00 days	n.r	BMI < 17.5, weight loss, life-threatening physical complications, suicide risk, chronic failure to benefit from outpatient treatment	Multidisciplinary: occupational therapists, social workers, physical education instructors, consultant physician, liaison with psychiatrists
5	Mander et al. [34]	n.r	48.80 days	20.5% employed, 35.9% in job training, 20.4% unemployed	n.r	Psychotherapists
6	Pemberton et al. [35]	n.r	n.r	n.r	BMI < 16	n.r
7	Zuchova et al. [36]	n.r	n.r	n.r	n.r	Psychiatrist, group facilitator
8	Hofer et al. [37]	Home, institution/therapeutic living situation, peripheral hospital	49.50 days	n.r	n.r	Psychiatrist, general clinician, dietician, psychologist
9	Schlegl et al. [38]	n.r	91.79 days	n.r	BMI ≤ 17.5	Medical doctors, psychologists
10	Andrade et al. [39]	n.r	n.r	n.r	n.r	Interdisciplinary project: psychologist, general clinician, dietician, nurse, psychiatric consultant
11	Hessler et al. [40]	n.r	91.22 days	n.r	BMI ≤ 17.5	Medical doctors, psychologists
12	Marzola et al. [41]	n.r	36.20 days	n.r	n.r	Psychiatrists, clinical psychologists, nurses, dietitian and internal medicine physician
13	Chatelet et al. [42]	Day care center, ambulatory, none	72.90 days	53.4% students, 14.56% employed, 10.68% unemployed, 23.3% disabled	Low BMI, weight loss, food intake, electrolytes, alcohol/drug	Psychotherapist, physiotherapist, dietician; multidisciplinary team
14	Dittmer et al. [43]	Outpatient care, day hospital, re-admission, communities	Group 1: 101.46 days; group 2: 99.82 days	n.r	Weight loss, BMI < 15 and > 13, medical complications, family/social factors, failure in an outpatient or day-hospital setting, purging or compulsive exercise, low insight, marked psychiatric comorbidity	TAU group: multimodal approach, psychiatric and general medical treatment; HEB group: clinical psychologist and a sports therapist
15	Gjoertz et al. [44]	n.r	Group 1: 96.70 days; group 2: 62.20 days	51.7% students, 22.6% disabled, 12.8% unemployed	n.r	Dietitians and nurses supervising the meals
16	Guarda et al. [45]	n.r	38.84 days	n.r	n.r	Psychotherapist, nurse
17	Guinhut et al. [46]	n.r	36.90 days	n.r	n.r	Physicians, psychiatrists, a clinical psychologist, a dietician, a physiotherapist, nurses and nursing assistants
18	Yamazaki et al. [47]	n.r	n.r	n.r	BMI < 18.5	n.r
19	Smith et al. [48]	n.r	Group 1: 4.29 weeks; group 2: 16.43 weeks	88% reported: 39.4% employed, 37.9% students, 22.7% unemployed	n.r	Psychiatrists, psychologists, dieticians, social workers, nurses and occupational therapists
20	Dauty et al. [49]	n.r	42.00 days	n.r	n.r	PMR physician, psychiatrist, physiotherapist, sports instructor, dietician, nurse
21	Young et al. [50]	n.r	25.53 days	n.r	BMI < 16	Psychiatrists and psychologists
22	Fouladi et al. [51]	n.r	Group 1: 4–10 weeks; group 2: 1–7 weeks	n.r	n.r	n.r
23	Funayama et al. [52]	n.r	n.r	n.r	n.r	Psychiatrist
24	Hellwig-Walter et al. [53]	n.r	n.r	n.r	n.r	n.r
25	Hemmingsen et al. [54]	Transfer to a specialized psychiatric unit or discharge to outpatient setting	41.00 days	n.r	Life threatening weight loss	Psychiatrist, somatic consultations, nurse
26	Riedlinger et al. [55]	n.r	9.50 weeks	n.r	n.r	Multidisciplinary: psychologist, clinician, nurse, therapists (music and art)
27	Sjögren et al. [56]	n.r	n.r	n.r	n.r	Multidisciplinary: physician, psychiatrist, psychologist, nurses, dietician, physiotherapist

N	Authors	BMI at admission (kg/m^2^)	Delta BMI (kg/m^2^)	Target BMI (kg/m^2^)	Refeeding protocol and nutrition
1	Carter et al. 2011 [30]	14.80	n.r	BMI ≥ 20	n.r
2	Money et al. 2011 [31]	14.70	n.r	n.r	n.r
3	Davies et al. 2012 [32]	Group 1: 14.64; group 2: 13.91	Group 1: 1.30, group 2: 1.80	n.r	n.r
4	Long et al. 2012 [33]	14.06	5.46	BMI 20	Use of NGT, assisted meals in group (after phase 1), energy intake from 1500 kcal/day to 2000 kcal/day (week 2) and to 2500 kcal (week 3 on). In case of weight gain under 1 kg/week: bed rest, use of protein drinks, locked bathroom and observation
5	Mander et al. 2013 [34]	14.94	1.25	n.r	n.r
6	Pemberton et al. 2013 [35]	n.r	n.r	n.r	n.r
7	Zuchova et al. 2013 [36]	Group 1: 15.03; group 2: 15.56	n.r	n.r	n.r
8	Hofer et al. 2014 [37]	13.70	1.30	n.r	1.2% parenteral nutrition, 13.9% NGT. Patients at risk for refeeding syndrome: day 1–3 10 kcal/kg, then slow increase to 15 kcal/kg + prophylactic electrolyte supplementation; day 4–6 15–20 kcal/kg; day 7–10 20–30 kcal/kg. Body weight measured daily up to day 6, then twice a week. Oral nutritional supplements
9	Schlegl et al. 2014 [38]	14.56	n.r	BMI ≥ 18	Expected weight gain 700 g per week, monitorized twice a week. In case of failure of weight gain: increase of food intake and monitoring during meal times, administration of high caloric fluids or feeding through nasal tube
10	Andrade et al. 2017 [39]	15.00	n.r	n.r	3 meals + 3 snacks, 1700 kcal
11	Hessler et al. 2019 [40]	n.r	2.54	n.r	Normal portions three times per day, mealtime support, free access to cafeteria/supermarket/lavatories
12	Marzola et al. 2019 [41]	14.16	0.81	n.r	Use of NGT, 3 meals + 2 snacks, assisted, in group
13	Chatelet et al. 2020 [42]	14.80	2.30	n.r	NGT if goal not achieved in 2 days. Nutrition stepwise approach: 50% of individual basic caloric needs at day 1, 75% at day 3, 100% at day 6. Second week: 300 kcal, 600 kcal and 1000 kcal added every 3 days. Third week: extra 1000 kcal per day on top of the basic needs
14	Dittmer et al. 2020 [43]	14.98	2.88	n.r	TAU: graduated exercise, + 700/1,000 g per week, biweekly weight checks, exercise contract, supervised meals 3 times per day. HEB: 8 sessions (twice per week) to reduce the excessive quantity of the exercise and the compulsive quality of the exercise
15	Gjoertz et al. 2020 [44]	14.30	2.70	n.r	3 meals + 3 snacks, assisted, in group, toilets locked for 2 h, weight target 1 kg/w, nutrition stepwise approach. No direct supplementing if a patient does not finish his/her plate. Oral replacement therapy in case of vitamin or electrolyte deficiencies
16	Guarda et al. 2020 [45]	n.r	n.r	BMI ≥ 19	Expected weight gain 1.4 kg/week, calories advanced to 3500–4000 kcal/day, close nursing observation and meal support
17	Guinhut et al. 2021 [46]	12.20	1.60	n.r	Initial supplementation with vitamins, phosphorus and trace elements. In patients with BMI < 13 enteral nutrition via NGT during the first 48 h. Initial enteral caloric intake 10 kcal/kg. Objective of caloric intake 30 kcal/kg/day
18	Yamazaki et al. 2021 [47]	13.58	n.r	n.r	Expected weight gain 1 kg/week, usage of supplementary enteral nutrition
19	Smith et al. 2021 [48]	n.r	n.r	n.r	n.r
20	Dauty et al. [49]	13.80	1.00	n.r	> 500 g per week**,** weight monitoring twice a week, NGT at entry > 1500 kcal × 36 gg approximately, dietary consultations once per week, supervised meals
21	Young et al. [50]	12.70	n.r	n.r	n.r
22	Fouladi et al. [51]	14.60	5.33	n.r	3200–3400 kcal at discharge
23	Funayama et al. [52]	13.40	n.r	n.r	Mainly oral nutritional rehabilitation, intravenous feeding sometimes used, less frequently NGT. Initial caloric prescription 600–1400 kcal/day, increased by 200 kcal/day. Maximum caloric intake 3000 kcal/day
24	Hellwig-Walter et al. [53]	14.72	n.r	n.r	n.r
25	Hemmingsen et al. [54]	13.10	n.r	n.r	Weight restoration 2–3% per week, meals supervised by nurses, supplemental nutrition if meal not consumed. If fail to reach weekly weight gain, increase of 600 kJ in diet and supervised rest in a seated position (30—60 min). Light physical activity permitted. If excessive urge to exercise, observation
26	Riedlinger et al. [55]	n.r	n.r	n.r	Oral nutritional rehabilitation with supervised meals and weight contract
27	Sjögren et al. [56]	14.85	n.r	n.r	Multidisciplinary therapy with supervised meals, weight monitored once per week. Five meals per day. Approximately 1 kg weight increase per week

N	Authors	Motivation	Referral by emergency room	Setting	Pharmacological treatment	Psychotherapy	Mortality (percentage)
1	Carter et al. 2011 [30]	n.r	n.r	Specialist ED unit	n.r	n.r	n.r
2	Money et al. 2011 [31]	n.r	n.r	Specialist ED unit	n.r	yes (CREST)	n.r
3	Davies et al. 2012 [32]	n.r	n.r	Two specialist ED units	n.r	CREST, CBT, CAT, Schema Therapy, group work, family work	n.r
4	Long et al. 2012 [33]	yes	n.r	Specialist ED unit	n.r	yes (CBT and PDP) + family education	n.r
5	Mander et al. 2013 [34]	yes	n.r	Psychiatric unit	n.r	yes (CBT with IP elements, twice a week) + group therapy	n.r
6	Pemberton et al. 2013 [35]	n.r	n.r	Specialist ED unit	n.r	n.r	n.r
7	Zuchova et al. 2013 [36]	n.r	n.r	Specialist ED unit	n.r	yes (group CRT)	n.r
8	Hofer et al. 2014 [37]	yes	n.r	Internal medicine unit	n.r	yes	0.00%
9	Schlegl et al. 2014 [38]	yes	n.r	Specialist ED unit	n.r	yes (CBT once or twice per week) + group therapy	n.r
10	Andrade et al. 2017 [39]	yes	yes	Specialist ED unit	only for comorbidities	yes (CBT), family involvement in autonomization of the patient	n.r
11	Hessler et al. 2019 [40]	n.r	n.r	Specialist ED unit	n.r	yes (CBT, once or twice a week) + group therapy	n.r
12	Marzola et al. 2019 [41]	yes	n.r	Specialist ED unit	n.r	yes (PDP, thrice per week) + weekly psychoeducational groups + parental counseling	n.r
13	Chatelet et al. 2020 [42]	n.r	n.r	Specialist ED unit	n.r	yes	n.r
14	Dittmer et al. 2020 [43]	yes	n.r	Specialist ED unit	n.r	yes (HEB arm) + problem solving groups (TAU arm), once or twice a week	n.r
15	Gjoertz et al. 2020 [44]	n.r	n.r	Specialist ED unit	yes (antipsychotics 51.75%, antidepressants 46.4%, anxiolytics 84.3%)	n.r	n.r
16	Guarda et al. 2020 [45]	n.r	n.r	Specialist ED unit	n.r	group psychotherapy, family meetings	n.r
17	Guinhut et al. 2021 [46]	n.r	yes	Specialist ED unit	n.r	n.r	1.40%
18	Yamazaki et al. 2021 [47]	n.r	n.r	Psychiatric unit	n.r	n.r	n.r
19	Smith et al. 2021 [48]	n.r	n.r	Specialist ED unit	n.r	n.r	n.r
20	Dauty et al. [49]	n.r	yes (intensive care)	Physical medicine/rehabilitation	n.r	n.r	n.r
21	Young et al. [50]	n.r	n.r	Medical care unit	yes (antidepressants 40%, anticonvulsants 12%, benzodiazepines 12%, antipsychotics 8%)	n.r	n.r
22	Fouladi et al. [51]	n.r	n.r	Specialist ED unit	n.r	n.r	n.r
23	Funayama et al. [52]	n.r	n.r	Neuropsychiatric unit	n.r	n.r	n.r
24	Hellwig-Walter et al. [53]	n.r	n.r	Psychosomatic medicine and psychotherapeutic ward	yes (antidepressants 31.58%)	n.r	n.r
25	Hemmingsen et al. [54]	n.r	n.r	Nutrition unit	yes	n.r	n.r
26	Riedlinger et al. [55]	n.r	n.r	Specialist ED unit	n.r	yes (multimodal psychotherapeutic approach) + groups	n.r
27	Sjögren et al. [56]	n.r	n.r	Specialist ED unit	yes (antidepressants 31%, antipsychotics 18%, anxiolytics 16%	no	n.r

N	Authors	Test	Main findings	Quality rating
1	Carter et al. 2011 [30]	ED psychopathology: EDE, EDEQ; general psychopathology: BDI, RSES, PI, BSI	Fear of gaining weight is a valid diagnostic criterion for AN, as it seems to differentiate between full and partial cases in relation to both general and specific psychopathology	4
2	Money et al. 2011 [31]	n.r	CREST proves to be beneficial in an inpatient setting, with patients generally responding positively to its simple and cooperative style	5
3	Davies et al. 2012 [32]	n.r	Enhancements in 'cold' cognition are attained shortly, while addressing emotion-processing challenges may take longer. The CREST group demonstrates larger effect sizes in emotion-processing tasks	3
4	Long et al. 2012 [33]	ED psychopathology: MRAS, EDI, BSQ, OANQ, ABOS; general psychopathology: BSI, CFSEI, HDL	Individuals with chronic AN require a multidisciplinary treatment focused on readiness for treatment and therapeutic alliance	5
5	Mander et al. 2013 [34]	No ED psychopathology testing reported; general psychopathology: SCL-90-R, URICA-S, SACiP	The URICA-S maintenance scale can serve as a useful tool for identifying potential relapses	5
6	Pemberton et al. 2013 [35]	n.r	Management strategies employed by some members of staff may maintain the symptoms of EDs	4
7	Zuchova et al. 2013 [36]	n.r	Group-based CRT can be incorporated into the therapeutic program, with the participants responding positively to it	3
8	Hofer et al. 2014 [37]	n.r	The severity and occurrence of complications during the replenishment phase in inpatients with AN can be kept to a minimum	4
9	Schlegl et al. 2014 [38]	ED psychopathology: EDI; general psychopathology: BSI, BDI	Treating AN with inpatient care is successful in achieving weight restoration	5
10	Andrade et al. 2017 [39]	n.r	The duration of illness significantly influences outcome and prognostic features	4
11	Hessler et al. 2019 [40]	ED psychopathology: SIAB-S; no general psychopathology testing reported	Different elements of weight history (highest weight, weight suppression, weight elevation, lowest weight, age at past weight and years since past weight) may be linked to various aspects of the ED	4
12	Marzola et al. 2019 [41]	ED psychopathology: ANSOCQ, EDEQ; general psychopathology: BDI, STAI, EQ‐5D‐VAS, WAI-SR	Baseline motivation to change is linked to therapeutic alliance at the time of discharge independently of other variables	5
13	Chatelet et al. 2020 [42]	n.r	It is important to start an intensive initial nutrition at admission to promote weight gain	5
14	Dittmer et al. 2020 [43]	ED psychopathology: SIAB-EX, CES, CET, EDEQ; general psychopathology: BSI, BDI, DERS	HEB is effective in reducing compulsive exercise among patients with atypical AN	4
15	Gjoertz et al. 2020 [44]	n.r	The use of a clinical guideline demonstrates significant effectiveness in promoting weight gain through intensive refeeding procedures while also maintaining safety	5
16	Guarda et al. 2020 [45]	ED psychopathology: EDEQ; no general psychopathology test reported	A behaviorally focused, integrated, meal-based treatment, aimed at achieving rapid weight gain, is considered acceptable by most of the patients	4
17	Guinhut et al. 2021 [46]	n.r	AN hospitalized patients present frequent medical complications and psychiatric comorbidity It is then crucial to provide specialized and multidisciplinary care	4
18	Yamazaki et al. 2021 [47]	n.r	Diets with higher levels of carbohydrate are associated with the development of refeeding hypophosphatemia in inpatients with AN	5
19	Smith et al. 2021 [48]	ED psychopathology: EDE, EDEQ; general psychopathology: BDI	Patients considered resistant to inpatient treatment differ in ED psychopathology and depressive symptoms from those with good outcomes at initial admission	4
20	Dauty et al. [49]	n.r	Initiating controlled physical activities early during hospitalization for severe AN does not compromise the effectiveness of intensive refeeding	3
21	Young et al. [50]	ED psychopathology: MAEDS, BCQ; general psychopathology: BABS, CIA	Future studies should investigate variations in the intensity of delusions and assess whether focusing on delusional beliefs improves the effectiveness of treatment for individuals with AN	5
22	Fouladi et al. [51]	n.r	Inpatient treatment leads to modifications in gut microbial metabolism and induces consistent alterations in the composition of gut microbial communities among patients with AN	4
23	Funayama et al. [52]	n.r	Nadir levels of hematological cells in inpatients with AN can be predicted in case of the restrictive type and infectious complications	5
24	Hellwig-Walter et al. [53]	ED psychopathology: EDEQ; general psychopathology: PHQ, EPS, DERS	During treatment, alterations in IGF-I levels are linked to both BMI and leptin. Age seems to influence how IGF-I responds to underweight	4
25	Hemmingsen et al. [54]	ED psychopathology: EDI; general psychopathology: TAS20, BDI, HADS, PSS10	The levels of cortisol in blood and urine remain stable from admission to discharge in patients with severe AN	4
26	Riedlinger et al. [55]	ED psychopathology: EDI; general psychopathology: PSQ, GSRS, GAD-7	Providing patients with information about gastrointestinal symptoms during the process of weight rehabilitation can contribute to adherence to treatment	5
27	Sjögren et al. [56]	ED psychopathology: EDEQ, EDI; general psychopathology: MDI, SCL92	Inpatient treatment for AN is linked to a decrease in feelings of depression	4

*DSM-IV* Diagnostic and Statistical Manual of mental disorders Fourth Edition, *DSM-5* Diagnostic and Statistical Manual of mental disorders Fifth Edition, *ICD* International Classification of Diseases and Related Health Problems 10th Revision, *ED* eating disorder, *ANR* Anorexia Nervosa Restricting Type, *ANBP* Anorexia Nervosa Binge-Purging Type, *AAN* Atypical Anorexia Nervosa, *F* female, *M* male, *BMI* body mass index, *TAU* Treatment As Usual, *HEB* Healthy Exercise Behavior, *PMR* Physical Medicine and Rehabilitation, *NGT* nasogastric tube, PDP, Psychodynamic Psychotherapy, *CBT* Cognitive Behavioral Therapy, *CRT* Cognitive Remediation Therapy, *CAT* Cognitive Analytic Therapy, *IP* Interpersonal Psychotherapy, *CREST* Cognitive Remediation and Emotion Skills Training, EDE, Eating Disorder Examination, *EDEQ* Eating Disorder Examination Questionnaire, *BDI* Beck Depression Inventory, *STAI* State Trait Anxiety Inventory, *EQ‐5D‐VAS* EuroQoL Quality of Life Scale Visual Analogue Scale, *WAI‐SR* Working Alliance Inventory‐Short Revised, *ANSOCQ* Anorexia Nervosa Stages of Change Questionnaire, *EDI* Eating Disorder Inventory, *BSI* Brief Symptom Inventory, *MRAS* Morgan–Russell Assessment Schedule, *BSQ* Body Shape Questionnaire, *OANQ* Overcoming Anorexia Nervosa Questionnaire, *ABOS* Anorectic Behavior Observation Scale, *CFSEI* Culture-Free Self-Esteem Inventory, *HDL* Health & Daily Living Form, *SCL-90-R* Symptom-Checklist- 90-Revised, *URICA-S* University Rhode Island Change Assessment Scale-short form, *SACiP* Multiperspective Assessment of General Change Mechanisms in Psychotherapy, *SIAB-S* Structured Inventory for Anorexic and Bulimic Disorders for DSM-IV and ICD-10—Self-Rating, *SIAB-EX* Structured Expert Interview for Anorexic and Bulimic Syndromes according to DSM-IV and ICD-10, *CES* Commitment to Exercise Scale, *CET* Compulsive Exercise Test, *DERS* Difficulties in Emotion Regulation Scale, *RSES* Rosenberg Self-Esteem Scale, *PI* Padua Inventory, *GAD-7* Generalized Anxiety Disorder 7, *PHQ* Patient Health Questionnaire, *PSQ* Perceived Stress Questionnaire, *GSRS* Gastrointestinal Symptom Rating Scale, *TAS-20* Toronto Alexithymia Scale, *HADS* Hospital Anxiety and Depression Scale, *PSS-10* Perceived Stress Scale 10, *EPS* Emotional Processing Scale, *BABS* Brown Assessment of Beliefs Scale, *MAEDS* Multifactorial Assessment of Eating Disorder Symptoms, *CIA* Clinical Impairment Assessment, *BCQ* Body Checking Questionnaire, *MDI* Major Depression Inventory, *SCL-92* Hopkins Symptom Checklist, *CRT* Cognitive Remediation Therapy, *CREST* Cognitive Remediation and Emotion Skills Training, *HEB* Healthy Exercise Behavior, *IGF* Insulin-like Growth Factor, *n.r.* data not reported

The authors (F.T., M.M., P.L., N.D., and M.P.) also reviewed the data to identify the primary aspects pertinent to hospitalization: ultimately, nine key themes were selected as the main outcomes, drawing from both the major factors explored in the existing literature and the methodology employed in the previous work by Suárez-Pinilla and colleagues [21]. The 2015 paper identified five treatment areas: antipsychotics, antidepressants, psychotherapy, nutrition, and "others". We retained similar themes (pharmacotherapy, psychotherapy, and nutrition) while incorporating additional areas of particular interest in the analysis of hospitalization practices (motivation, clinical team, setting, admission/discharge criteria, follow-up, and psychometric assessment). Afterward, the authors determined the percentage of studies referencing a specific theme, described the subthemes investigated in each article and identified any areas lacking sufficient coverage. Oversight and guidance for the study were provided by G.A.D.

## Results

This review comprises a selection of 27 studies, encompassing a total of 3501 patients. Data about the different populations of patients analyzed in the single studies are available in Table 1. While not every study provided details on the gender of the patients, 97.55% of the population in 24 studies were female [31, 33–50, 52–56]. Employment status was reported in five studies [30, 40, 42, 44, 48], revealing that about 47.5% were students, 25.67% were employed, and 15.72% were unemployed. The average age of patients in the 26 studies that reported this information was 27.22 years [30–34, 36–56] and the mean length of inpatient stay in 18 studies was 59.35 days [30, 33, 34, 37, 38, 40–42, 44–51, 54, 55]. Diagnosis subtype was specified in only 20 of the referenced studies [30, 33, 37–50, 52–55], with about 57.16% corresponding to the restrictive type of AN, and 40.73% to the binge-purging type. The mean duration of the disease was 8.50 years [30, 32–34, 36–39, 41–44, 46, 48–50, 52–56]. Among the 24 studies defining BMI at admission, the mean BMI value was 14.20 [30–33, 36–54, 56].

In this sample of 27 studies, specific dimensions of hospitalization in AN were explored. The subsequent sections provide a detailed examination of the nine themes identified, shedding light on the clinical team, psychotherapeutic approaches, motivation factors, protocols, nutrition practices, pharmacological treatments, admission and discharge criteria, follow-up procedures, and psychometric assessments. A synthesis of the results is shown in Table 2.

**Table 2:** Hospitalization themes

Theme	Percentage (%)	Subthemes
1. Clinical team	81.48	Psychiatrist: 13/27 Psychologist: 11/27 Nurse: 10/27 General Clinician: 7/27 Dietician: 9/27 Physiotherapist: 4/27 Occupational therapist/social worker: 2/27
2. Psychotherapeutic framework	51.85	Cognitive Behavioral Therapy: 6/27 Psychodynamic Therapy: 2/27 Cognitive Remediation and Emotion Skills Training: 2/27 Cognitive Remediation Therapy: 1/27 Cognitive Analytic Therapy: 1/27 Schema Therapy: 1/27
3. Motivation	25.93	Use of validated questionnaires: 3/27
4. Treatment setting	100	Eating Disorders Unit: 19/27 Psychiatry Unit: 3/27 Internal Medicine Unit: 2/27 Physical Medicine and Rehabilitation Unit: 1/27 Psychosomatic and Psychotherapeutic Wards: 1/27 Nutrition Unit: 1/27
5. Nutrition and refeeding	66.67%	Details about meals: 13/27 Nasogastric Tube Feeding: 10/27 Details about toilet locking/supervised rest: 4/27 Details about target weight gain: 8/27 Weight monitoring frequence: 4/27 Details about caloric intake: 7/27 Details about physical exercise: 4/27
6. Psychopharmacological therapy	22.22	Antipsychotics: 3/27 Antidepressants: 4/27 Anxiolytics: 3/27 Anticonvulsants/mood-stabilizers: 1/27 Pharmacotherapy not detailed: 2/27
7. Admission/discharge criteria	40.74	Admission criteria: 9/27 - BMI: 8/27 - Additional criteria (other than BMI): 4/27 Discharge: 5/27 - BMI: 5/27
8. Follow-up	14.81	Outpatient care: 4/27 Day Hospital: 2/27 Residential treatment: 2/27 Transfer to a specialized unit: 1/27
9. Psychometric assessment	51.85	Questionnaires for ED: 13/27 - EDE/EDEQ: 7/27 - EDI: 5/27 Questionnaires for general psychopathology: 12/27 - BDI: 5/27 - BSI: 4/27

The table illustrates the nine themes selected (Theme) and the respective percentages of studies that address these themes out of the total papers included in the review (Percentage). In the third column, the most relevant data and aspects for every factor (Subthemes) are taken into account, with the corresponding number indicating the studies considering the subthemes out of the total 27 included in our review (expressed as a fraction).

*BMI* body mass index, *ED* eating disorder, *EDE* Eating Disorder Examination, *EDEQ* Eating Disorder Examination Questionnaire, *EDI* Eating Disorder Inventory, *BDI* Beck Depression Inventory, *BSI* Brief Symptom Inventory

### Clinical team

Out of the 27 studies, 13 cited the presence of psychiatrists [33, 36, 37, 39, 41, 43, 46, 48–50, 52, 54, 56], 11 of psychologists [37–41, 43, 46, 48, 50, 55, 56], ten of nurses [39, 41, 44–46, 48, 49, 54–56], seven of general clinicians [33, 37, 39, 41, 43, 54, 56], nine of dieticians [37, 39, 41, 42, 44, 46, 48, 49, 56], four of physiotherapists [42, 46, 49, 56], two of occupational therapists and social workers [33, 48]. Only 12 studies detailed the presence of a multidisciplinary group [33, 37, 39, 41–43, 46, 48, 49, 54–56].

### Psychotherapeutic theoretical framework

In terms of methodology, among the studies that provided details (14 in total) [31–34, 36–43, 45, 55] the majority employed Cognitive Behavioral Therapy (CBT) (six studies) [32–34, 38–40], followed by Psychodynamic Therapy (two studies) [33, 41]. Additional studies outlined the application of Cognitive Remediation and Emotion Skills Training [31, 32], Cognitive Remediation Therapy [36], Cognitive Analytic Therapy and Schema Therapy [32], and incorporated components of Interpersonal Psychotherapy [34]. These methodologies were employed either independently or as adjunctive approaches. Only five studies explicitly clarified the frequency of psychological sessions, with three conducting it once or twice per week [38, 40, 43], one twice a week [34], and one claiming three times a week [41]. Nine of the studies incorporated therapy groups [32, 34, 36, 38, 40, 41, 43, 45, 55], and five studies involved family through meetings, education or counseling [32, 33, 39, 41, 45].

### Motivation

Out of the 27 studies, only seven addressed the issue of motivation [33, 34, 37–39, 41, 43]. Three studies utilized questionnaires, namely The Anorexia Nervosa Stages of Change Questionnaire (ANSOCQ) [41], Overcoming Anorexia Nervosa Questionnaire (OANQ) [33] and the University Rhode Island Change Assessment Scale (URICA) [34], to assess motivation. The remaining studies generically recommended including motivation to change in the treatment and emphasized the importance of encouraging the patient. One study indicated the evaluation of motivation upon admission, wherein therapists were instructed to assess and rate it on a scale ranging from 0 to 4 [38].

### Inpatient treatment protocols

Among the reviewed studies, 18 provided information on the mean duration of inpatient stay, indicating 59.35 days [30, 33, 34, 37, 38, 40–46, 48–51, 54, 55]. Two studies mentioned that the patients could be referred from the emergency room [39, 46], while another stated that they were transferred from an intensive care unit [49]. All the studies specified the type of ward, with 19 being specialist EDs inpatient units [30–33, 35, 36, 38–46, 48, 51, 55, 56], three Psychiatry units [34, 47, 52], two Internal Medicine units [37, 50], one Physical Medicine and Rehabilitation unit [49], and one Nutrition unit [54]. Additionally, one study was conducted in two integrated Psychosomatic and Psychotherapeutic wards [53].

### Reefeding and nutrition

Of the total of studies, 18 provided some information about refeeding practices. Regarding oral nutrition, two studies outlined three meals per day [40, 43], two reported three meals plus two snacks [41, 56], and other two specified three meals plus three snacks [39, 44]. Meals were often taken in groups with assistance from nurses or dieticians during meals, according to 11 studies [33, 38, 40, 41, 43–45, 49, 54–56], although this was not recommended by two studies at the beginning of the treatment [33, 38]. In case of incomplete feeding or insufficient weight gain, four studies reported administering direct supplementation with an oral or enteral hypercaloric solution [33, 38, 42, 54]. One study specified the absence of direct supplementing in case of incomplete meals [44].

In the total of 27 distinct studies, ten implemented the use of a nasogastric tube (NGT) [33, 37, 38, 41, 42, 44, 46, 47, 49, 52]. In one study NGT was utilized within the first 48 hours if the BMI was less than 13, as part of a refeeding protocol [46]. Another study reported using NGT if nutritional objectives were not met within two days [42], and yet another if there was insufficient weight gain [38]. In a different study, NGT was used by default with an intake at the entry of >1500 Kcal/day, gradually reducing by 250 Kcal per week if the weekly objective of weight gain was reached [49]. Additionally, two studies specified routine electrolyte supplementation [37, 46].

Two studies mentioned locking toilets after meals [33, 44], with one specifying a 2-h lock [44]. Conversely, in one study, access to toilets was free [40]. In one study, all meals were followed by supervised rest in a seated position lasting from 30 to 60 min [54]. For three of the studies, the weekly weight gain goal was 1000 g [44, 47, 56], while the remaining studies had varied goals, such as 700 g per week [38], one between 700 and 1000 g [43], one 1400 g week [45], one more than 500 g per week [49]. In one study, if a patient failed to achieve 2% weekly weight gain, the energy value of the menu was increased, typically by 600 kJ [54].

Only five studies specified weight monitoring twice a week [43, 49], while another reported weight measurement once per week [56]. In another study body weight was measured daily up to day 6, then twice a week [37].

Discussing caloric intake, various studies proposed different approaches. Two studies recommended a gradual increase, starting with 50% of individual basic energy needs on day 1, reaching 100% on day 6, and adding 300 kcal every 3 days in the second week [42, 44]. Basic energy needs were defined as 35 kcal/kg/d for adults. Another study opted for a non-hypercaloric diet (1700 kcal/day) to achieve a minimum weight compatible with health [39]. A different approach involved an initial intake of 1500 kcal/day, increasing to 2000 kcal in the second week and further to 2500 kcal from the third week onward [33]. Three studies specified final caloric intakes: one at 3500-4000 kcal/day [45], another at 3200-3400 kcal/day [51], and a third setting the maximum at 3000 kcal/day [52]. Additionally, the latter study mentioned an initial prescription of 600–1400 kcal/day, typically increased by about 200 kcal daily.

Four studies specified that physical exercise was permitted in a controlled manner, not compromising the efficiency of intensive refeeding [33, 43, 49, 54]. Specifically, in one study, light physical activity could be performed, but for patients with an excessive urge to exercise, observation by a trained nurse was up to one-to-one supervision 24 hours a day [54]. In another study patients were first helped by physiotherapy, subsequently by physical education staff to engage at the advised activity level according to their physical health status (group sport/swimming to provide social activity and aerobic exercise in the maintenance phase) [33]. In another study, patients treated with treatment as usual (TAU) took part in graduated exercise therapy and were advised to elaborate an “exercise contract” with their therapist, while Healthy Exercise Behavior (HEB) Intervention patients engaged in a program aimed at reestablishing a “healthy” exercise behavior, reducing the compulsive quality of the exercise and re-experiencing social interaction and relaxation [43].

### Psychopharmacological therapy

Among the analyzed inpatient treatments, only six addressed pharmacotherapy [39, 44, 50, 53, 54, 56], indicating that patients could receive antipsychotics (three studies) [44, 50, 56], antidepressants (four studies) [44, 50, 53, 56] and/or anxiolytics (three studies) [44, 50, 56]. Only one study mentioned the utilization of anticonvulsants as mood stabilizers [50]. One study cited the use of pharmacotherapy without providing specific details [54]. Another study simply stated that six out of 19 patients were taking antidepressants [53]. Notably, one study emphasized that pharmacotherapy was not considered a significant part of the therapeutic strategy, for its ineffectiveness in treating EDs symptoms; however, pharmacotherapy could be important for managing other comorbidities [39].

### Admission and discharge criteria

Out of the 27 samples, 25 specified that the diagnosis of AN would be based on criteria from the ICD-10 (International Classification of Diseases 10th Revision), DSM-5 (Diagnostic and Statistical Manual of Mental Disorders, Fifth Edition), or DSM-IV (Diagnostic and Statistical Manual of Mental Disorders, Fourth Edition) [30–35, 37–50, 52–56]. However, only eight studies utilized BMI as a criterion for admission, with cut-off values specified in three studies as less than 17.5 [33, 38, 40], in two studies as less than 16 [35, 50], and one each for less than 18.5 [47], less than 15 [43], and “low BMI at admission” [42]. Four studies mentioned additional general criteria, including increased/rapid/life-threatening weight loss, minimal food intake for several days, electrolyte imbalances, alcohol/drug abuse, life-threatening physical complications, suicide risk, chronic failure to benefit from outpatient treatment, family/social factors impeding recovery, pronounced purging or compulsive exercise, low insight, marked psychiatric comorbidity, and severity and chronicity of the illness [33, 42, 43, 54].

Five studies employed BMI as a discharge criterion, three specifying a value equal to or greater than 20 [30, 33, 48], one equal to or greater than 19 [45], and another equal to or greater than 18 [38]. The mean delta BMI, from 13 studies that provided this information, is 2.32 [32, 33, 36–38, 41–44, 46, 48, 49, 51]. The studies did not mention any other specific discharge criteria besides BMI.

Only two studies addressed mortality: one reported a mortality rate of 0% [37], while the other mentioned a rate of 1.47% [46].

### Follow-up

Five studies specified the characteristics of the follow-up phase, with four outlining that patients could transition to ambulatorial outpatient care [33, 42, 43, 54], two continuation to Day Hospital care [42, 43], two mentioning residential treatment [37, 43], and one indicating that patients might be transferred to a specialized psychiatric unit [54]. The remaining 22 studies did not provide details on the follow-up procedure.

### Psychometric assessment

To assess ED psychopathology, 13 studies employed psychological scales. The majority (seven studies) used the Eating Disorder Examination (EDE, EDE-Q) [30, 41, 43, 45, 48, 53, 56], while five studies employed the Eating Disorder Inventory (EDI) [33, 38, 54–56]. Other scales included the Multiaxial Assessment of Eating Disorders Symptoms (MAEDS) [50], Anorexia Nervosa Stages of Change Questionnaire (ANSOCQ) [41], Morgan–Russell Assessment Schedule (MRAS) [33], Body Shape Questionnaire (BSQ) [33], Body Checking Questionnaire (BCQ) [50], Overcoming Anorexia Nervosa Questionnaire (OANQ) [33], Anorectic Behaviour Observation Scale (ABOS) [33], Commitment to Exercise Scale (CES) [43], Compulsive Exercise Test (CET) [43], and the Structured Inventory for Anorexic and Bulimic Disorders for DSM-IV and ICD-10 (SIAB) [40, 43].

Regarding general psychopathology, 12 studies utilized psychological scales [30, 33, 34, 38, 41, 43, 48, 50, 53–56]. Among these, five used the Beck Depression Inventory (BDI) [30, 38, 41, 48, 54], and four employed the Brief Symptom Inventory (BSI) [30, 33, 38, 43]. Other scales included the State–Trait Anxiety Inventory (STAI) [41], EuroQoL EQ‐5D‐VAS [41], Working Alliance Inventory‐Short Revised (WAI-SR) [41], Culture-Free Self-Esteem Inventory (CFSEI) [33], Health & Daily Living Form (HDLF) [33], Symptom-Checklist-90-Revised (SCL-90-R) [34] and Hopkins Symptom Checklist (SCL-92) [56], the University of Rhode Island Change Assessment Scale-short version (URICA-S) [34], Multiperspective Assessment of General Change Mechanisms in Psychotherapy (SACIP) [34], Rosenberg Self-Esteem Scale (Rosenberg) [30], Generalized Anxiety Disorder 7 (GAD-7) [55], Patient Health Questionnaire (PHQ) [53], Perceived Stress Questionnaire (PSQ) [55], Gastrointestinal Symptom Rating Scale (GSRS) [55], Toronto Alexithymia Scale (TAS-20) [54], Hospital Anxiety and Depression Scale (HADS) [54], Perceived Stress Scale 10 (PSS-10) [54], Emotional Processing Scale (EPS) [53], Difficulties in Emotion Regulation Scale (DERS) [43, 53], Clinical Impairment Assessment (CIA) [50], Brown Assessment of Beliefs Scale (BABS) [50], Padua Inventory (PI) [30] and Major Depression Inventory (MDI) [56].

## Discussion

The data of the present systematic review on a large sample confirm that adults affected by AN are usually young (mean 27.22 years) and with an average BMI of 14.20 that falls below the threshold for extreme severity as defined by DSM-5 [57]. The average length of stay in hospital is protracted but shorter than what was reported in literature from the 1990s onwards (59.35 days in the present review compared to 76.40 days in previous literature), confirming that, probably due to changes in the organization of health systems and/or the advancement of research, the length of stay is decreasing [58]. Furthermore, the average duration of the illness is 8.5 years, suggesting it is a long-lasting condition that often does not respond to initial treatments. This finding is consistent with the recent meta-analysis by Solmi and colleagues [4].

More in detail, this comprehensive analysis explores various facets of AN treatment, from refeeding practices and psychotherapeutic approaches to pharmacotherapy and diagnostic criteria. Despite advances in understanding AN, the varied descriptions and heterogeneous information in the studies seem to reflect the challenges in achieving consensus and uniformity within the field [59, 60]. This challenge is also grounded in the complex task of translating guidelines into real-world practice. However, this heterogeneity not only underscores the complexity of AN, but also contributes to a lack of common information, compromising the ability to establish standardized protocols and reducing the generalizability of findings across different treatment settings.

In our review, approximately 81.48% of the studies provided insights into the clinical team, while 51.85% referenced using a psychotherapeutic model. Motivation was addressed in 25.93% of the studies, and all specified the treatment setting. Nutrition and refeeding protocols were detailed in 66.67% of the studies, while pharmacological therapy was mentioned in a small number of papers (22.22%). Admission or discharge criteria were described in 40.74% of the studies, and follow-up procedures were discussed in 14.81%. Additionally, 51.85% of the studies incorporated tests for assessment of the ED or general psychopathology. Approximately 50.62% of the studies, on average, presented information regarding key aspects of treatment, highlighting a lack of sufficient details about inpatient settings in ED treatments. This may hinder the possibility of a uniform description of interventions, pointing out a significant gap in the current research literature.

### Treatment setting

The majority of guidelines, including the latest from the American Psychiatric Association [16], recommend the presence of a multidisciplinary team. However, less than half of the analyzed studies align with this indication and provide detailed information about the treatment team [33, 37, 39, 41–43, 46, 48, 49, 54–56]. Typically, this team should include psychiatrists, dieticians, nurses, clinical psychologists, and internal medicine physicians, with regular team meetings and supervision emphasized [12, 17]. However, the composition of these teams varied widely in the studies, and the presence of specific professionals was not consistently outlined. Since scientific articles also contribute to the establishment of new treatment centers, not specifying this data in detail is problematic. For example, the presence of a psychiatrist was explicitly mentioned in only 48.15% of the studies [33, 36, 37, 39, 41, 43, 46, 48–50, 52, 54, 56], possibly assumed to be implicit. The significance of nurses was highlighted in 37.04% of the studies [39, 41, 44–46, 48, 49, 54–56], not recognizing enough the unique challenges they face in caring for hospitalized patients with EDs, including meal supervision, managing physical activity, and understanding psychopathological features [61]. Only 33.33% of the studies [37, 39, 41, 42, 44, 46, 48, 49, 56] acknowledged the role of dietitians, even though the American Dietetic Association itself emphasized the pivotal role of nutrition intervention as an integral component of team treatment for AN [62]. Finally, since medical stabilization and nutrition is a central element during hospitalization, the role of the doctor specialized in clinical nutrition is very little recognized and described, even though it is so essential. This flaw could arise from the fact that the majority of studies are carried out by mental health researchers. Adjunctive treatment recommendations were infrequently made, with only a few studies mentioning the presence of a physiotherapist (14.81%) [42, 46, 49, 56], or an occupational therapist (7.41%) [33, 48] among the total of studies. This limited acknowledgment of rehabilitation professionals overlooks the crucial role they play in addressing physical and functional aspects during inpatient treatment [63, 64].

### Psychotherapy

Although important for addressing psychological mechanisms, cognitive processes, behavioral patterns and providing emotional support, only some specific psychotherapies are recommended during hospitalization [1]. 51.85% of the studies referenced in the present review utilized some form of psychotherapy during inpatient treatment. Cognitive Behavioral Therapy [32–34, 38–40] and Psychodynamic Therapy [33, 41], employed by the majority of studies, are endorsed by several articles and reviews [65–68] and APA guidelines [16], which recommend ED–focused psychotherapy for adults with AN. Family Based Treatment, recommended for younger patients [69], was not prevalent, as our review excluded pediatric subjects. Individual psychotherapeutic management during acute renourishment can offer psychoeducation and support [70]; however, the initiation of psychotherapy in this acute phase should be tailored to the patient's medical stability and readiness, as recommended by the APA in 2023 [16]. Literature on psychotherapy during hospitalization is, in fact, ambivalent, with some studies considering it not effective in the acute phase, due to emaciation and negativism [71], and others evaluating it as promising and highlighting a better harmonization of treatments from inpatient to outpatient care [72]. This can partially explain in our sample the scarce number of studies specifying a psychotherapeutic approach. Two studies in our review also cited Cognitive Remediation and Emotion Skills Training (7.41%%) [31, 32] as well as Cognitive Remediation Therapy (3.70%) [36], which are explored in novel research [73–75]. However, the studies that used a specific theoretical framework rarely detailed the weekly frequency of the sessions (18.52%) [34, 38, 40, 41, 43], and a few cited therapy groups (33.33%) [32, 34, 36, 38, 40, 41, 43, 45, 55] and parental counseling (18.52%) [32, 33, 39, 41, 45]. Furthermore, it is essential to consider that variations in adopting psychotherapeutic practices may stem from differences in the types of wards included in the studies. These practices are likely more prevalent in specialized units such as ED, general Psychiatry, or Psychosomatic units, as opposed to Internal Medicine or Physical Rehabilitation units. This datum seems to be confirmed by our review, in which 56.52% of the psychiatric units implement some form of psychotherapy, compared to 25% of the remaining settings. Again, future studies need to better define what clinical teams do and how they do it to psychologically support the patient. In fact, beyond formalized psychotherapies or more extensive psychological help in therapies-as-usual, defining how to support patients during hospitalization is mandatory.

### Psychometric and motivation assessment

Although the use of an ED rating scale is not imperative to quantify eating and weight control behaviors, numerous patient and clinician-rated scales, along with screening tools for EDs, have been developed and validated [16, 76]. Surprisingly in assessing ED psychopathology, only 13 studies (48.15%) [30, 33, 38, 40, 41, 43, 45, 48, 50, 53–56] in our review employed psychological scales, with the most frequently used tests being the Eating Disorder Examination Questionnaire [77, 78] (25.93%) [30, 43, 45, 48, 53, 56, 79] and the Eating Disorder Inventory [78] (18.52%) [33, 38, 54–56]. However, it is highlighted that in half of the studies there is no interest in evaluating through scores the improvement or the cognitive and behavioral aspects of the disease, which are maintenance factors and necessary to work on for recovery. It would be useful if a minimum set of psychometric scales were always included in studies on hospitalization. Even more so, there is a consensus in acknowledging EDE and EDI as the most reliable and broadly applicable ones for AN-specific symptomatology.

Conversely, the exploration of motivation in the context of AN was limited, with only seven studies addressing the topic (25.93%) [33, 34, 37–39, 41, 43]. Notably, only one study (3.70%) mentioned the implementation of a motivational interview before admission [38], despite the recognition that poor motivation is associated with treatment dropout and negative outcomes [80, 81]. This points to a gap in the practice, given that motivation in AN is influenced by both psychological and biological factors inherent to the illness [82, 83]. Also, motivation should be regarded as a pivotal factor, given its direct correlation with the severity of the condition. The greater the severity, the higher the likelihood of hospitalization, underscoring the imperative to address and enhance motivation in such cases.

### Refeeding and nutrition

Nutritional treatment is the primary intervention in AN as well as in other EDs [4]. While oral nutrition is the preferred method in the studies analyzed for the review, being mentioned in the total of studies, there is no clear indication of the quantity, quality, and supervision of meals, resulting in some disparity between the various studies. This reflects a lack of standardized refeeding protocols regarding oral nutrition, contrary to the literature which suggests the implementation of specific supervised meal programs along with supervision training [18], and an insufficient definition of the importance of using liquid nutritional supplements [84]. Also, toilet-locking durations [33, 40, 44] or supervised rest after meals [54] are rarely mentioned (14.81%) and vary across studies.

In the treatment landscape of AN, refeeding for weight restoration is crucial, but practices lack standardization, relying on clinical expertise [70]. Oral nutrition is recommended as the primary treatment, while NGT feeding has limited impact on normalizing food intake or diversifying the diet [18] and should be considered a short-term intervention before transitioning to oral intake [16] in severely malnourished patients [18] or when fear of weight gain poses challenges to oral nutrition [85]. However, only ten studies (37.04%) in our review discussed NGT as a choice of treatment [33, 37, 38, 41, 42, 44, 46, 47, 49, 52], usually as a secondary strategy after an initial attempt with entirely oral nutrition (29.63%) [33, 37, 38, 41, 42, 44, 47, 52]. Thus, the criteria for its implementation and the categorization of NGT as a primary or secondary treatment are infrequently specified in our sample and exhibit variation across studies. Also, aspects of tolerance, acceptability, and psychological education of the patient are not cited, although they should be integral components of the programs [86].

According to the consensus on weight gain for patients with AN [16], only 25.93% of the studies in our review indicate a weekly weight gain target within the range of 0.5–1.5 kg [38, 43–45, 47, 49, 56]. This aligns with the established recommendation to balance concerns about refeeding syndrome [87] and patient tolerance [88], even though some researchers suggest further research on faster weight restoration [45]. Overall, it is important to emphasize that not only the target weight, but also the pattern of weight gain (trajectory), which considers fluctuations and is highly personalized, is a crucial aspect [89, 90]. Unfortunately, our sample of studies did not explore this aspect so crucial for patients and clinicians.

Regarding caloric intake, diverse studies proposed different approaches, with two recommending a gradual increase starting from 50% of individual basic energy needs [42, 44] and another suggesting an initial intake of 1500 kcal/day [33]. These findings (11.11%) align with the current trend of using higher initial caloric prescriptions and faster rates of renourishment under close medical monitoring, with electrolyte correction as needed [18, 91, 92]. In contrast, one study (3.70%) mentioned an initial prescription of 600–1400 kcal/day [52], reflecting historical concerns about refeeding syndrome [16]. Final caloric intakes are specified to be between 3000–4000 kcal/day in 11.11% of the studies [45, 51, 52], in line with literature recommending higher energy intake in individuals with a high rise in energy expenditure [93, 94]. Conversely, another study (3.70%) opted for a non-hypercaloric diet (1700 kcal/day) to achieve a minimum weight compatible with health [39]. These data highlight a persistent variance in clinical practice regarding caloric prescription, likely influenced by clinical judgment and personal preference. Unfortunately, none of the studies mention the importance of discussing this crucial aspect [95] with the patient.

Interestingly, while there is limited literature on physical therapy in AN suggesting the potential benefits of aerobic exercise and yoga [96], four studies (14.81%) in our research delved into this aspect [33, 43, 49, 54], with one implementing a Healthy Exercise Behavior intervention [43]. Current knowledge suggests that incorporating physical activity during the refeeding of patients with AN is safe and beneficial for restoring body composition and bone mineral density, as well as improving mood and anxiety [64].

### Admission, discharge, and follow-up

The admission and discharge criteria for AN lack a strict definition in the studies under review. Criteria for admission are described only in 33.33% of the studies and include low BMI, rapid or life-threatening weight loss, minimal food intake, electrolyte imbalances, alcohol/drug abuse, physical complications, suicide risk, chronic failure to benefit from outpatient treatment, family/social factors, pronounced purging or compulsive exercise, and marked psychiatric comorbidity [33, 35, 38, 40, 42, 43, 47, 50, 54]. However, these norms only partially correspond to the more defined criteria outlined in the most recent APA guidelines, as the latter include specific levels of heart rate (< 50 bpm), blood pressure (< 90/60 mmHg), glucose (< 60 mg/dL), electrolytes, temperature (< 36 °C), rapidity of weight change (> 10% in 6 months), and ECG abnormalities (QTc > 450 ms) [16]. This suggests that in clinical practice, hospitalization is often influenced not only by standard guidelines criteria, but also by environmental factors, concurrent psychiatric symptoms, and “clinical wise”. While this individualized approach may be relevant for each patient, the lack of consistency could lead to a situation where treatment varies significantly between centers. Moreover, the APA defines BMI < 15 as a possible criterion for hospitalization but cautions against relying solely on this parameter for severity assessment and admission decisions, considering factors such as the rate of weight loss, even at non-extreme BMI.

Additionally, only five studies (18.52%) in our review employed BMI as a discharge criterion [30, 33, 38, 45, 48], and the mean delta BMI from the 13 studies that reported it is 2.32 [32, 33, 36–38, 41–44, 46, 48, 49, 51]. The latter represents a significant outcome and seems to reflect the effectiveness of hospitalization, despite variations in practices. However, it is important to note the difference in the length of stay of the studies included (see Table 1) and recognize that BMI alone does not provide a comprehensive assessment of treatment efficacy and effectiveness. For example, weight gain trajectory was proposed as a relevant outcome [97]. Also, apart from BMI, the studies did not mention any other specific discharge criteria. This indicates a lack of information about other parameters, which are probably considered outcome criteria, such as the stabilization of medical conditions, compliance with the dietary plan, familial and social factors, and the treatment of comorbidities [79, 98]. We also have to consider that in treating AN, reliance on DSM criteria alone for discharge decisions poses significant challenges: many patients discharged based on these criteria (i.e., weight for partial remission) remain at high risk for relapse due to unresolved psychological or behavioral issues. This situation often results in a 'revolving door' phenomenon, where patients repeatedly enter and exit treatment [79].

Addressing the importance of a well-defined care plan, the NICE guidelines stress the significance of articulating how patients will be discharged and reintegrated into community-based care [10]. However, our sample revealed that the transition of AN patients to various care settings (e.g., Day Hospital, outpatient treatment, residential center) was explored in only five studies (18.52%) [33, 37, 42, 43, 54]. The challenges associated with the termination of inpatient treatment like discontinuity of care [20] and the high post-discharge relapse rates [99] underscore the need for a clearer definition of interventions following hospitalization.

### Psychopharmacological therapy

As echoed in both guidelines and clinical practice, pharmacotherapy is not the primary treatment for AN, and, although frequently used, is associated with low recovery rates [4]. In particular, limited evidence supports the use of antidepressants for weight gain during nutritional rehabilitation; however, the established practice involves an integrated approach to medications for managing specific symptoms and comorbidities [9]. These compounds, especially selective serotonin reuptake inhibitors or SSRIs, are therefore commonly prescribed [100–102], accounting for more than 35% of the patients in our review [44, 50, 53, 56] and reflecting the relevance of depressive comorbidity in AN [103]. Also, the off-label use of antipsychotics, notably olanzapine and aripiprazole, is frequent in AN treatment [104–107], reflected in about 20% of the patients in this review [44, 50, 53, 56]. In some cases of AN, mood stabilizers may be prescribed, typically in the presence of mood or personality disorders in comorbidity [108], although they are not considered a first-line treatment for AN, constituting only 3% of patients in our review [50]. Benzodiazepines and anxiolytics are frequently used for the temporary treatment of anxious symptoms and sleep–wake cycle disturbances [109], accounting for more than 25% of patients in the present study [44, 50, 56]. While pharmacotherapy is commonly used in real-world practice, it is then important to note that the majority of the included studies (77.78%) omitted important data about the presence of pharmacologic treatment, and the six that reported lacked relevant information about the specific compounds used, the rationale for the treatment, and data about treating comorbid conditions.

## Conclusion

Hospitalization is a crucial phase in the treatment pathways for individuals with AN. In this setting they are encountered during the most acute, severe, and thus challenging conditions, making it essential to propose consistent and appropriate treatments in line with established guidelines. However, our review outlines the presence, in clinical practice, of a wide range of criteria, objectives, and treatment forms, posing a risk of inconsistency and deviation from evidence-based medicine.

Although the majority of studies cited some members of the clinical team, only less than half provided detailed information on a multidisciplinary team. The studies also showed significant variation in composition: specific professionals, such as psychiatrists and nurses, were inconsistently outlined. This contrasts with the knowledge that EDs are complex diseases that need treatment by a wide range of healthcare figures [110].

The therapeutic field also lacks uniformity, particularly in the implementation of psychotherapy, with no consensus on its necessity in the acute phase, a fragmentation of the data about the type of psychotherapeutic approach, and a notable deficiency in the focus on motivation within treatment. Few studies addressed pharmacotherapy, contrasting with its widespread use in clinical reality to treat comorbid symptoms. Moreover, the reports not only lack details on the medications used, but also on the treatment rationale, interactions with other proposed treatments, side effects, and patient adherence. It is also worth noting that the previous review from Suárez and colleagues [21] included greater detail regarding pharmacological treatments, while our work evidenced a lack of reporting on the same theme: this may potentially shed light on a sort of slowdown in advancements in this field during the last decade [111].

Furthermore, our review also highlights a lack of strict definitions for admission and discharge criteria, influenced by environmental factors and psychiatric symptoms. It is noteworthy to consider that while there may be criteria in practice, their absence in the studies suggests an underreporting or lack of clear treatment goals. Discharge parameters beyond BMI are rarely mentioned, indicating a potential gap in the identification of outcome criteria. Transition to various care settings post-hospitalization, crucial for patient care and relapse prevention, was explored in only five studies.

In terms of nutrition, oral rehabilitation is preferred, consistent with clinical guidelines. However, there is a disparity in the quantity, quality, and supervision of meals across studies. Also, although guidelines specify certain criteria for the introduction of nasogastric tubes and the target weight gain, there is a lack of standardized refeeding protocols and inconsistent mention of measures of behavioral control (e.g., toilet-locking duration, supervised rest).

In conclusion, our review exposes the incomplete and heterogeneous nature of descriptions surrounding AN inpatient treatments, from refeeding protocols to psychotherapeutic and pharmacotherapeutic approaches, outlining inconsistent reporting practices. While it is well established that in the real-world treatments may be different from the gold standards of international guidelines [112], it could be helpful, when presenting results for a scientific paper to offer a complete description of treatment settings, especially regarding inpatients units, and to implement the use of uniform reporting of the practices.

Future studies should comprehensively detail team composition, clinical orientation, pharmacological and nutritional treatments, admission criteria, discharge goals, and psychotherapy or rehabilitative interventions. A further effort in describing treatments in this complex field is commendable, also given the fact that EDs risk being considered a niche in actual research [113], and thus, individuals with ED risk receiving inconsistent and non-gold standard treatments.

Further studies should delve deeper into inpatient treatment approaches, elucidating the reasons for implementation and their advantages/disadvantages: this would enhance the medical community's understanding of alternative treatments, their benefits, and impacts, contributing to the development of universal guidelines for a more cohesive and scientifically supported treatment reality.

## Strengths and limits

The strengths of this review lie in its comprehensive examination of various aspects of AN inpatient treatments, providing insights into the diverse criteria, objectives, and treatment forms present in clinical practice. A potential limitation could be the heterogeneity among the included studies, which may impact the generalizability of findings. The variation in study designs, populations, clinical settings, and methodologies may limit the ability to draw uniform conclusions applicable across diverse settings and patient groups. Additionally, an inherent challenge lies in the ambiguity of whether the lack of certain data stems from them just not being described in papers, indicating potential hidden criteria that may align with established guidelines, or from the absence of use of criteria, resulting in patients receiving heterogeneous treatments among different centers.

### Supplementary Information

Below is the link to the electronic supplementary material.

Supplementary file1 (DOCX 44 KB)

Supplementary file2 (DOCX 20 KB)

Supplementary file3 (DOCX 31 KB)

## Data Availability

Datasets are available on request through contact with the corresponding author.
