# Supplementary material for: Inpatient treatments for adults with anorexia nervosa: a systematic review of literature
Source: Eat Weight Disord. 2024 May 20;29(1):38. doi: 10.1007/s40519-024-01665-5 (PMC11106202; doi:10.1007/s40519-024-01665-5)
Supplement: Supplementary file 1 — Supplementary file1 (DOCX 44 KB) [file 40519_2024_1665_MOESM1_ESM.docx]

**Inpatient treatments for adults with Anorexia Nervosa: a systematic review of literature**

Federica Toppino, Matteo Martini, Paola Longo, Inês Caldas, Nadia Delsedime, Raffaele Lavalle, Francesco Raimondi, Giovanni Abbate-Daga, Matteo Panero

**Identification of studies via databases and registers**

Records removed *before screening*:

Duplicate records removed (n = 14)

Records identified through database and register searching

(n = 846)

**Identification**

Records excluded by title and abstract

(n = 727)

Records screened

(n = 832)

Reports excluded:

Non adults (n = 49)

Non-hospital setting (n = 21)

General EDs (n = 8)

**Screening**

Reports assessed for eligibility

(n = 105)

Studies included in review

(n = 27)

**Included**
